# Supplementary figures and images for: The Impact of a Cypovirus on Parental and Filial Generations of Lymantria dispar L
Source: Insects. 2023 Nov 30;14(12):917. doi: 10.3390/insects14120917 (PMC10743831; doi:10.3390/insects14120917)

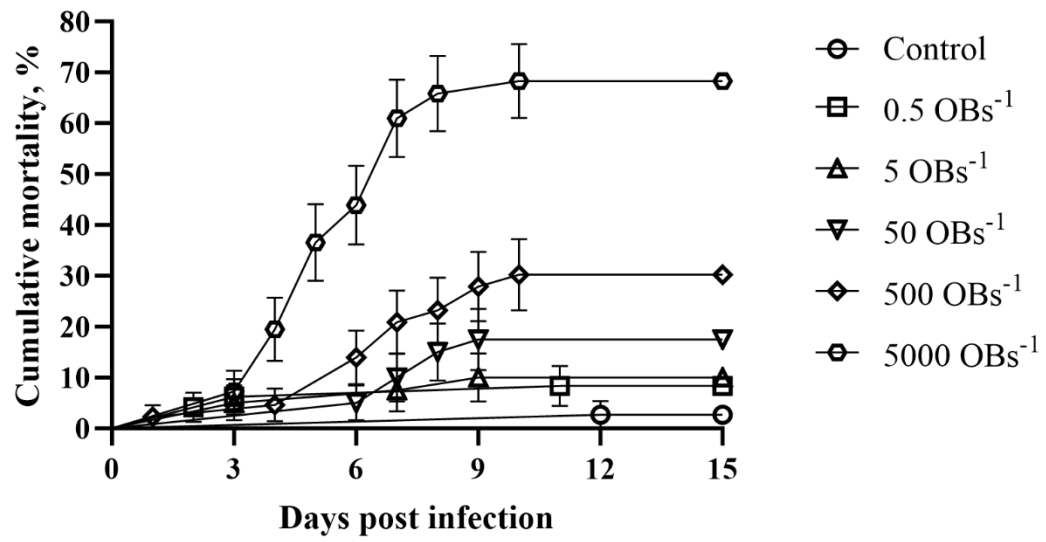

Figure S1. Mortality dynamics of parental *L. dispar* larvae after DsCPV1 infection

Supplement: Supplementary file 1 [file insects-14-00917-s001.zip › insects-2669585-supplementary.pdf]
